# Effects of Eucalyptus Essential Oil on Growth, Immunological Indicators, Disease Resistance, Intestinal Morphology and Gut Microbiota in *Trachinotus ovatus*

**DOI:** 10.3390/microorganisms13030537

**Published:** 2025-02-27

**Authors:** Ziyang Lin, Shengzhe An, Chuanpeng Zhou, Yaqi Chen, Zhenchuang Gao, Juan Feng, Heizhao Lin, Pengwei Xun, Wei Yu

**Affiliations:** 1 School of Fisheries, Xinyang Agriculture and Forestry University, Xinyang 464000, China; linziyang97@163.com (Z.L.); 17538578107@163.com (Y.C.); 13103976852@163.com (Z.G.); 2 Key Laboratory of Efficient Utilization and Processing of Marine Fishery Resources of Hainan Province, Sanya Tropical Fisheries Research Institute, Sanya 572426, China; chpzhou@163.com; 3 Key Laboratory of South China Sea Fishery Resources Exploitation & Utilization, Ministry of Agriculture and Rural Affairs, South China Sea Fisheries Research Institute, Chinese Academy of Fishery Sciences, Guangzhou 510300, China; shengzhean703@163.com (S.A.); jannyfeng@163.com (J.F.); linheizhao@163.com (H.L.); 4 Shenzhen Base of South China Sea Fisheries Research Institute, Chinese Academy of Fishery Sciences, Shenzhen 518121, China

**Keywords:** eucalyptus essential oil, immunological parameters, intestinal microflora, disease resistance, *Trachinotus ovatus*

## Abstract

Essential oils serve as potential additives that can enhance immune respons and disease resistance and regulate the gut microbiota of fish. Here, this research aims to identify the impacts of eucalyptus essential oil (EEO) on growth, liver antioxidative and immune parameters, resistance to *Streptococcus iniae*, intestinal morphology and gut microbiota in *Trachinotus ovatus*. All fish (initial weight: 26.87 ± 0.30 g) were randomly allocated to 12 floating cages (2.0 × 2.0 × 2.0 m^3^) with each cage containing 100 fish and fed for 30 days. Four diets were manufactured with the supplementation of varying levels of EEO (control and 5.0, 10.0, and 15.0 mL/kg) and were named CG, EEO1, EEO2 and EEO3, respectively. The results showed that EEO1 and EEO2 diets significantly increased WGR, thickness of the intestinal muscle layer, and the ratio of villus height to crypt depth (V/C), while decreasing the intestinal crypt depth of *T. ovatus* (*p* < 0.05). Significantly increased activities of SOD and CAT and significantly reduced MDA levels were present in the EEO1 and/or EEO2 groups (*p* < 0.05). Moreover, the mRNA levels of *nrf2*, *HO-1*, *GSH-Px*, *SOD*, *C4* and *GR* genes were significantly upregulated and the expression of *keap1* and *HSP70* genes were significantly downregulated within the EEO1 and EEO2 groups (*p* < 0.05). After challenge with *S. iniae B240703* for 24 h, the bacterial load for five organs in the EEO2 group was less than that in the CG group (*p* < 0.05). In addition, the fish fed EEO1 and/or EEO2 diets had significantly lower abundances of pathogenic bacteria (*Proteobacteria*, *Planctomycetota*, *Burkholderia-Caballeronia-Paraburkholderia*, *Pseudomonas* and *Blastopirellula*) and a higher beneficial bacteria proportion (*Firmicutes*) than those fed the CG diets (*p* < 0.05). In conclusion, a moderate dietary m EO level (5.0~10.0 mL/kg) improved the growth and gut morphology, promoted liver immune response, enhanced resistance to *S. iniae* and modulated the gut microbiota of *T. ovatus*.

## 1. Introduction

In recent years, intensive aquaculture has overly emphasized high stocking densities, which has led to reduced growth, poor immunity, increased disease outbreaks and substantial economic losses in farmed fish [1,2]. Antibiotics have been banned in aquaculture due to the environmental pollution, drug residues and increased bacterial resistance they cause [3], even though they are effective in treating fish diseases. Therefore, strengthening immunity and the capacity to resist diseases is now considered an important strategy for sustainable aquaculture.

Essential oils (EOs) are aromatic compounds derived from various parts of plants, and their main components include hydrocarbons, aldehydes, ketones, alcohols, phenols, ethers, terpenes, and phenolic esters [4,5]. There has been growing attention towards the application of EOs as a substitute for antibiotics in aquaculture in order to enhance the immunity of fish and managing disease outbreaks [6,7,8]. For instance, EO derived from bay laurel (*Laurus nobilis* L.) boosted growth and immune reactions, enhanced oxidation resistance, and regulated inflammatory pathways in *Oreochromis niloticus* [2]. Pepper-rosmarin (*Lippia sidoides* Cham.) EO promoted immunity and histomorphology in zebra fish [9]. Eucalyptus essential oil (EEO) is a flavescent liquid obtained by steam distillation of eucalyptus leaves [10,11]. The main component of EEO is 1,8-cineole, which accounts for 70–90% of EEO [12]. Several studies have confirmed that EEO is permitted for use as a dietary supplement because of its notable biological properties, which include anti-inflammatory, pest-repelling, and antioxidant qualities among others [13,14,15]. In sheep, supplementation with eucalyptus oil demonstrated a linear reduction in methane emissions, while also exhibiting no negative effects on gas production or the concentration of volatile fatty acids [16]. 1,8-cineole was reported to alleviate oxidative stress and inflammation in common carp subjected to copper and ammonia exposure [17,18]. Eucalyptus oil is also reported to be an environmentally friendly anesthetic in studies on European seabass (*Dicentrarchus labrax*) and meagre (*Argyrosomus regius*) [19]. In addition, research examining the effectiveness of eucalyptus extracts against virulent bacteria such as *Escherichia coli*, *Staphylococcus aureus* and *Klebsiella pneumoniae* in humans has been conducted [20,21,22,23]. A previous study found that essential oil of *Eucalyptus globulus* Labill. has antibacterial activity against seven strains of pathogenic bacteria in fish including *Edwardsiella tarda*, *Streptococcus iniae*, *S. parauberis*, *Lactococcus garviae*, *Vibrio harveyi*, *V. ichthyoenteri* and *Photobacterium damselae* [24]. Nevertheless, research examining the effects of eucalyptus oil on pathogenic bacteria in fish is still limited.

Golden pompano (*Trachinotus ovatus*) represents an economic species within deep-water cage culture in southern China, with aquaculture output reaching 292,263 tons [25]. The frequent occurrence of bacterial, parasitic and viral diseases threatens the sustainable development of aquaculture. The common diseases of *T. ovatus* overall include virus disease, bacterial disease and parasitic disease, which can cause serious harm to the growth and development of the fish and bring great economic loss to farmers. Research on the impact of EEO on fish, especially disease resistance against oceanic pathogens, has been relatively limited.

This objective of this research is to investigate the impact of EEO on growth, immunological parameters, resistance to *Streptococcus iniae*, intestinal morphology and gut microbiota in *T. ovatus*.

## 2. Materials and Methods

### 2.1. Diets and Fish

Four eucalyptus oil diets (Table 1) were prepared by mixing with a basic pellet diet. The basic diet was a kind of commercial diet produced by Guangdong Yuequn Marine Biotechnology Co., Ltd. (Jieyang, China) The four test diets were manufactured by supplementing graded levels of EEO (0, 5.0, 10.0, 15.0 mL/kg), which were named CG, EEO1, EEO2 and EEO3, respectively. To ensure the uniformity of EEO in the diets, water and double steamed wine were replaced with incremental levels of EEO. Double steamed wine was purchased from Guangdong Jiujing Distillery Co., Ltd. (Foshan, China). EEO was purchased from Jiangxi Hairui Natural Plant Co., Ltd. (Ji’an, China) The relative density and content of 1,8-cineole of EEO is, respectively, 0.859 and 70.19% according to the method of Pharmacopoeia of the People’s Republic of China 2020 Edition [26].

The EEO and wine were mixed, then water was added to dilute and mixed evenly. The mixed solution was sprayed on the surface of the diets by using a pressure sprayer to make the diets absorb the solution evenly in the mixing machine. All measures were taken to ensure uniform mixing of the EEO and the diet. Then, the mixed diets were sealed and stored in cold storage at −20 °C for 24 h. Diets were weighed according to daily feeding quantity, vacuum packed and stored at −20 °C until use.

Prior to the commencement of the feeding trial, 1200 fish were provided with basic diets. Fish (initial weight: 26.87 ± 0.30 g) were arbitrarily chosen and placed into twelve net cages (8 m^3^) containing 100 fish per cage. The diets were randomly distributed in these floating cages. Fish were administered feed twice daily at 7:00 and 17:00, with the feeding process conducted by hand until clear signs of satiation were observed. The test lasted 30 days.

### 2.2. Sampling

Following a night of fasting, the mass and number of fish from each cage were documented to assess weight growth rate (WGR) and survival rate. Three fish from each cage were sacrificed and liver and intestine tissues were obtained for the measurement of biochemical indicators and morphological indicators, respectively. An additional three fish from each cage were subjected to dissection to collect intestinal content for gut microbiota analysis.

### 2.3. Intestinal Morphology

For the middle gut specimens of experimental fish in groups CG, EEO1, EEO2, and EEO3, we conducted dehydration through a series of graded ethanol solutions followed by clarification in xylene. The embedding in paraffin wax and subsequent hematoxylin–eosin staining were handled by Sevier Biotechnology Co. (Wuhan, China). We evaluated the parameters (crypt depth, muscle layer thickness and villus height) of gut morphology using the Case Viewer software (V.2.4, Budapest, Hungary) provided by 3DHISTECH.

### 2.4. Antioxidant Enzyme Activities and Immune-Related Gene Expression in Liver

The liver specimens were subjected to homogenization in a cold homogenizing liquid. The top layer liquid was extracted following centrifuging the homogenate. The activities of SOD, CAT, MDA and T-AOC were tested utilizing kits (Nanjing JianchengBioengineering Institute, Nanjing, China) on a biochemical analyzer. RT-qPCR was conducted utilizing the SuperReal PreMix Plus (SYBR Green) (Vazyme, Nanjing, China) on a Roche LightCycler^®^ II 96 system (Roche, Basel, Switzerland) to measure the expression of the target genes. Primer sequences utilized for RT-qPCR are presented in Table 2. The β-actin gene of fish was used as a housekeeping gene. RT-qPCR was conducted as described in the Supplementary Materials.

### 2.5. Colonization of Streptococcus Iniae in Fish

*S. iniae B240703* was acquired from the South China Sea Fisheries Research Institute located in Guangzhou, China. At the conclusion of the experiment, ten fish from each cage were randomly selected and injected intraperitoneally with 200 μL of 1 × 10^8^ cfu/mL concentration of the *S. iniae B240703* strain. After being injected for 24 h, three fish were dissected and the brain, liver, spleen, kidneys, and head kidney tissues of each fish were surgically removed and subsequently placed into pure ethanol to extract DNA. This process was followed by the quantification of bacterial load utilizing absolute quantitative PCR techniques. The gyrB gene was targeted for the absolute quantification of *S. iniae B240703* (Table S1). The PCR reaction procedure was the same as in Section 2.4. qPCR was performed utilizing tissue-derived DNA as templates, and the quantification of bacterial copy numbers was achieved by correlating the CT values with the standard curve [30].

### 2.6. Intestinal Microbiota Analysis

The presence of gut microorganisms was identified via Origin-gene Biotechnology Co. Ltd. (Shanghai, China). Initially, the HiPure Stool DNA Mini Kit B was used to extract DNA of microbiota (D3141, Magen, Guangzhou, China). The V3 to V4 regions of 16S ribosomal RNA genes were amplified through forward primer 341F (5′-CCTACGGGNGGCWGCAG-3′) and reverse primer 805R (5′-GACTACHVGGGTATctaATCC-3′). Following PCR amplification, the DNA products were separated by electrophoresis, purified, and quantified. Next, the sequences were processed utilizing the Illumina MiSeq platform (Illumina, San Diego, CA, USA). Ultimately, the acquired sequences were analyzed through bioinformatics methodologies.

### 2.7. Data Analysis

The data are expressed as means ± SD derived from three replicates. All results underwent one-way analysis of variance (ANOVA), succeeded by Duncan’s multiple range tests. A *p*-value of less than 0.05 was deemed statistically significant. All assessments were conducted utilizing SPSS version 27.0 for the Windows operating system.

## 3. Results

### 3.1. Growth and Intestinal Morphology

**Figure 1:**
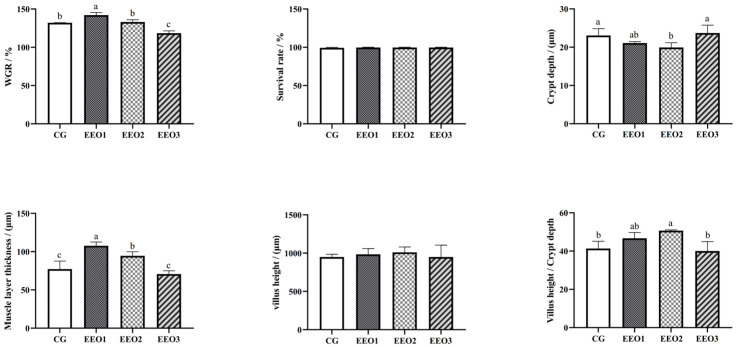

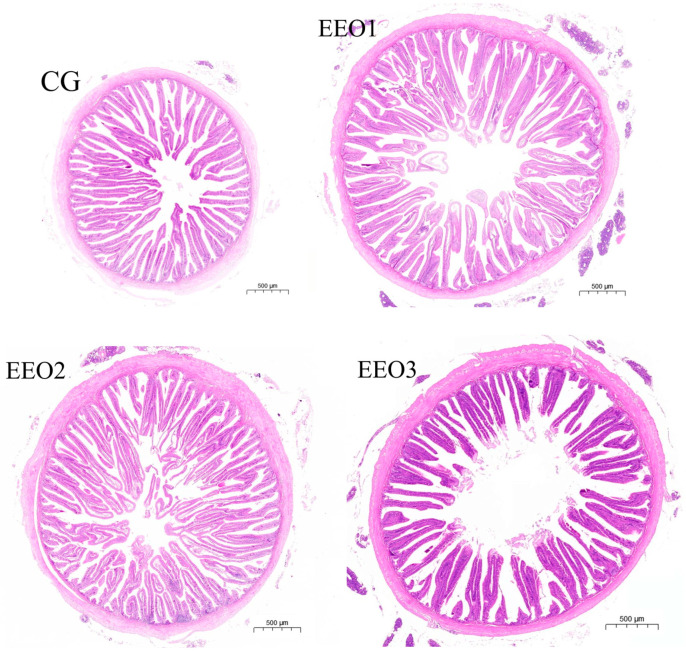
The growth performance and intestinal histology of *T. ovatus*. Each value represents mean ± SD (*n* = 3). Different letters above the bar represent significant differences among different groups (*p* < 0.05).

The findings are shown in Figure 1. Compared to the CG group, the WGR of fish fed EEO1 diets significantly increased (*p* < 0.05), while the fish in the EEO3 group showed a significant decrease in WGR (*p* < 0.05). Fish fed EEO1 and EEO2 diets emerged with markedly higher muscle layer thickness than those fed CG diets (*p* < 0.05). The crypt depth of fish in the EEO2 group was remarkably lower than of those in the CG group (*p* < 0.05). There were no statistically significant differences in villus height among the groups examined (*p* > 0.05). The EEO2 group demonstrated a markedly elevated V/C ratio in the intestine relative to the CG group (*p* < 0.05).

### 3.2. Hepatic Antioxidative Ability

**Figure 2:**
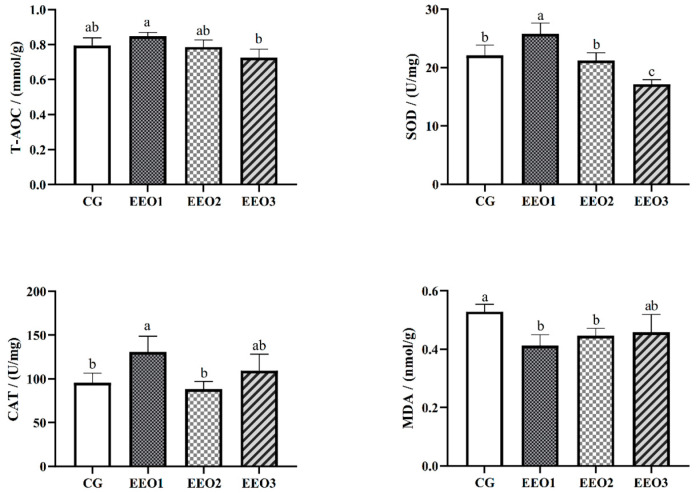
The hepatic antioxidative ability of *T. ovatus*. Each value represents mean ± SD (*n* = 3). Different letters above the bar represent significant differences among different groups (*p* < 0.05).

**Figure 3:**
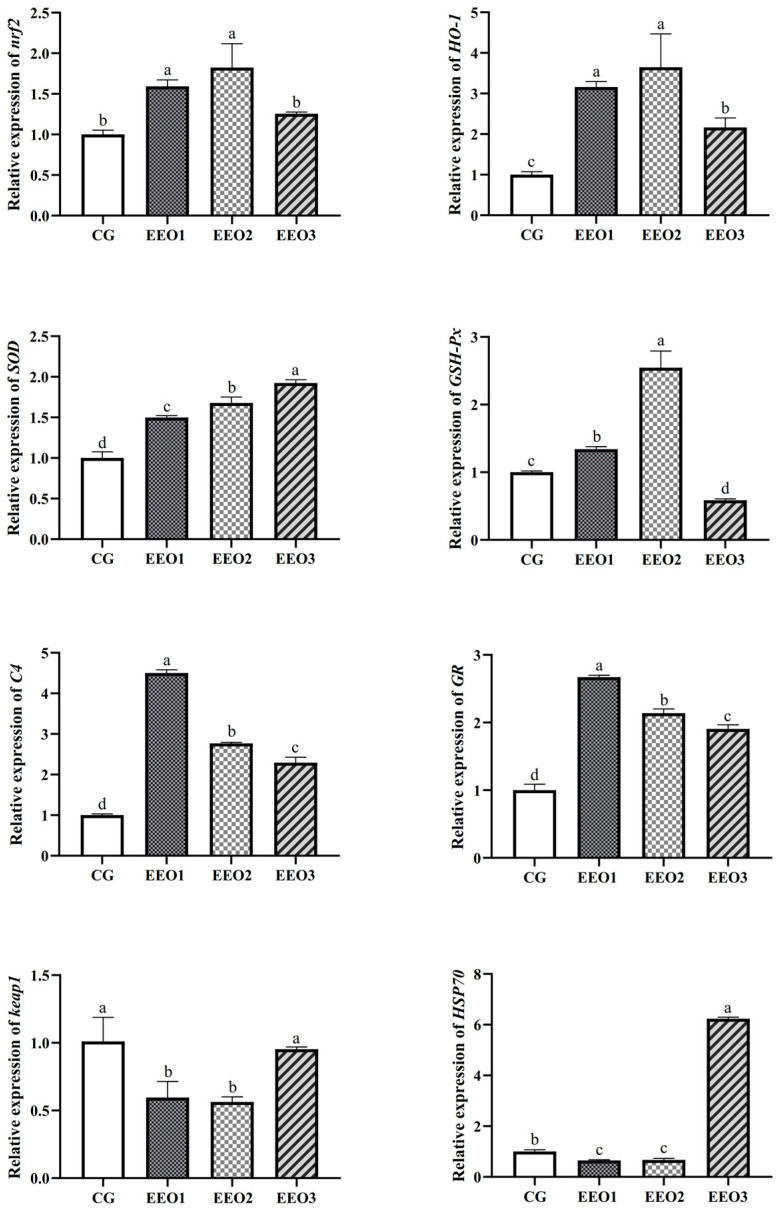
The hepatic immune response of *T. ovatus*. Each value represents mean ± SD (*n* = 3). Different letters above the bar represent significant differences among different groups (*p* < 0.05).

The levels of antioxidative enzymes are illustrated in Figure 2. The parameters of T-AOC, SOD and CAT presented a trend of first increasing and then decreasing with an increasing level of EO. The fish fed the EEO1 diet exhibited observably elevated SOD and CAT activities alongside a significantly reduced MDA level in comparison with those fed the CG diet (*p* < 0.05). The results of gene expression associated with oxidative stress are depicted in Figure 3. Compared to the CG group, EO supplement groups had markedly promoted expression levels of *HO-1*, *SOD*, *C4* and *GR* genes (*p* < 0.05). Moreover, the fish fed the EEO1 and EEO2 diets had significant upregulation in *nrf2* and *GSH-Px* genes while having significant downregulation in *keap1* and *HSP70* (*p* < 0.05). The mRNA levels of *GSH-Px* in the EEO3 group were remarkably downregulated and the expression of *HSP70* gene was remarkably upregulated compared to those in the CG group (*p* < 0.05).

### 3.3. Tissue Bacterial Load Following Challenge with S. iniae B240703

**Figure 4:**
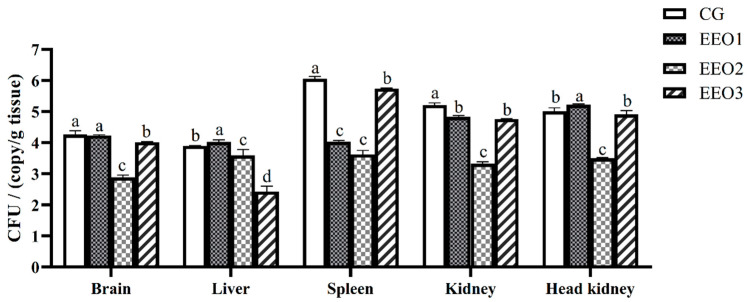
Bacterial load in *T. ovatus* specimens fed different diets 24 h after challenge with *S. iniae B240703* (dose 10^8^ CFU mL^−1^). Each value represents mean ± SD (*n* = 3). Different letters above the bar represent significant differences among different groups (*p* < 0.05).

According to the expression levels of the 16S rRNA *S. iniae B240703* gene, the bacterial load in the brain, liver, spleen, kidney and head kidney of infected subjects was assessed. The results are illustrated in Figure 4. Pathogen load analysis revealed that the bacterial loads in the spleen and kidney of the EO-supplement groups were significantly lower than those in the CG group 24 h after challenge (*p* < 0.05). The pathogen load in the brain and liver of the EEO2 and EEO3 group were lower than in the CG group (*p* < 0.05). The amount of *S. iniae* in all organs in the EEO2 group was less than that in the CG group at 24 h (*p* < 0.05).

### 3.4. Intestinal Microbiota

**Figure 5:**
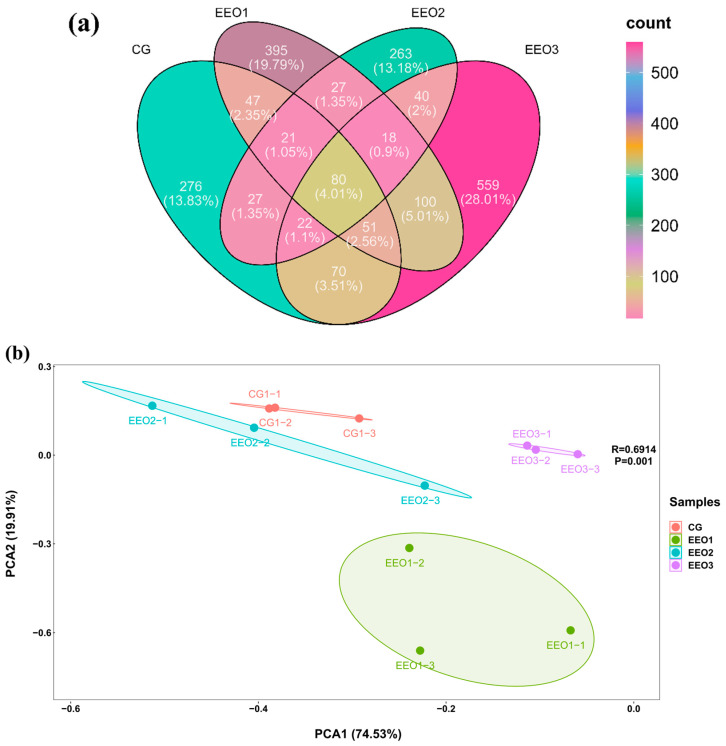
Diversity parameters of gut microorganism. (**a**) Venn diagram. (**b**) Beta diversity by Principal Component Analysis (PCA).

The α-diversity results are illustrated in Table 3. The fish fed EEO3 diets had evidently higher Observed_species, Shannon, Simpson, Chao1, Pielou_e and Ace than those fed CG, EEO1 and EEO2 diets (*p* < 0.05). A Venn diagram was created to analyze the similarities and differences of OTUs in each group (Figure 5a). There were a total of 1974 OTUs in the CG, EEO1, EEO2 and EEO3 groups. A total of 80 mutual OTUs were identified across the four groups. The distinct OTUs identified within the CG, EEO1, EEO2 and EEO3 groups were 276, 395, 263 and 559, respectively. The beta diversity of microorganisms was evaluated through the application of PCA (Figure 5b). The findings implied a distinct differentiation in the bacterial community structure among the four groups. Moreover, EEO2 and CG had a closer distance in comparison with EEO1 and EEO3 groups.

**Figure 6:**
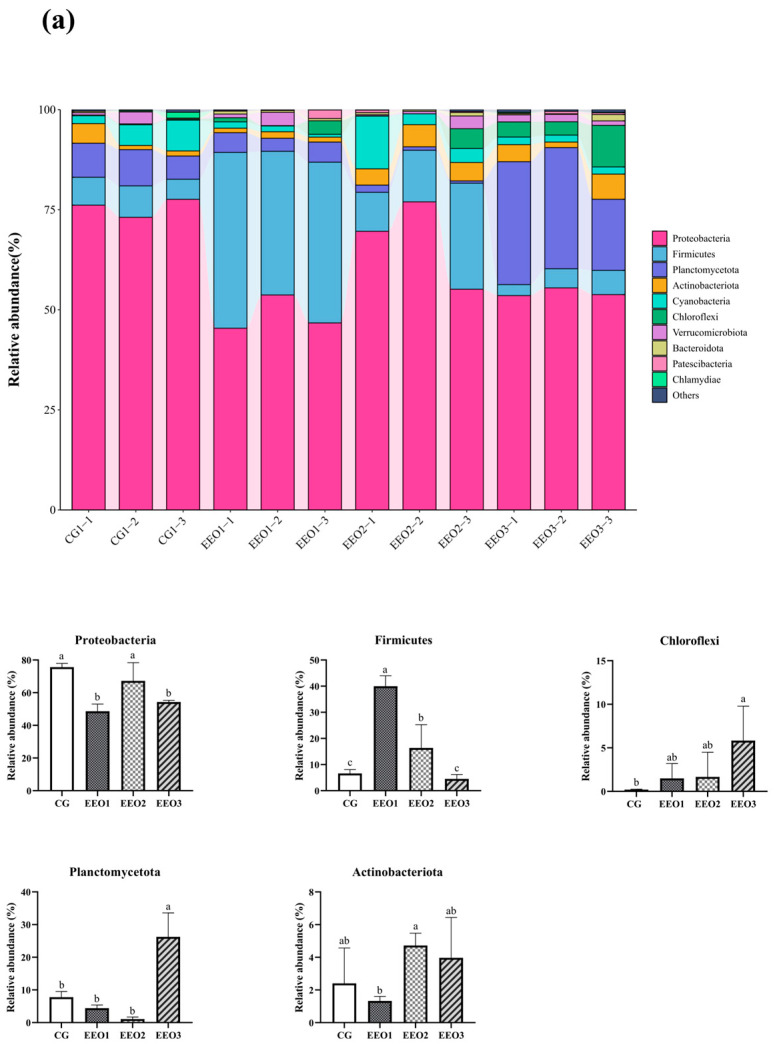

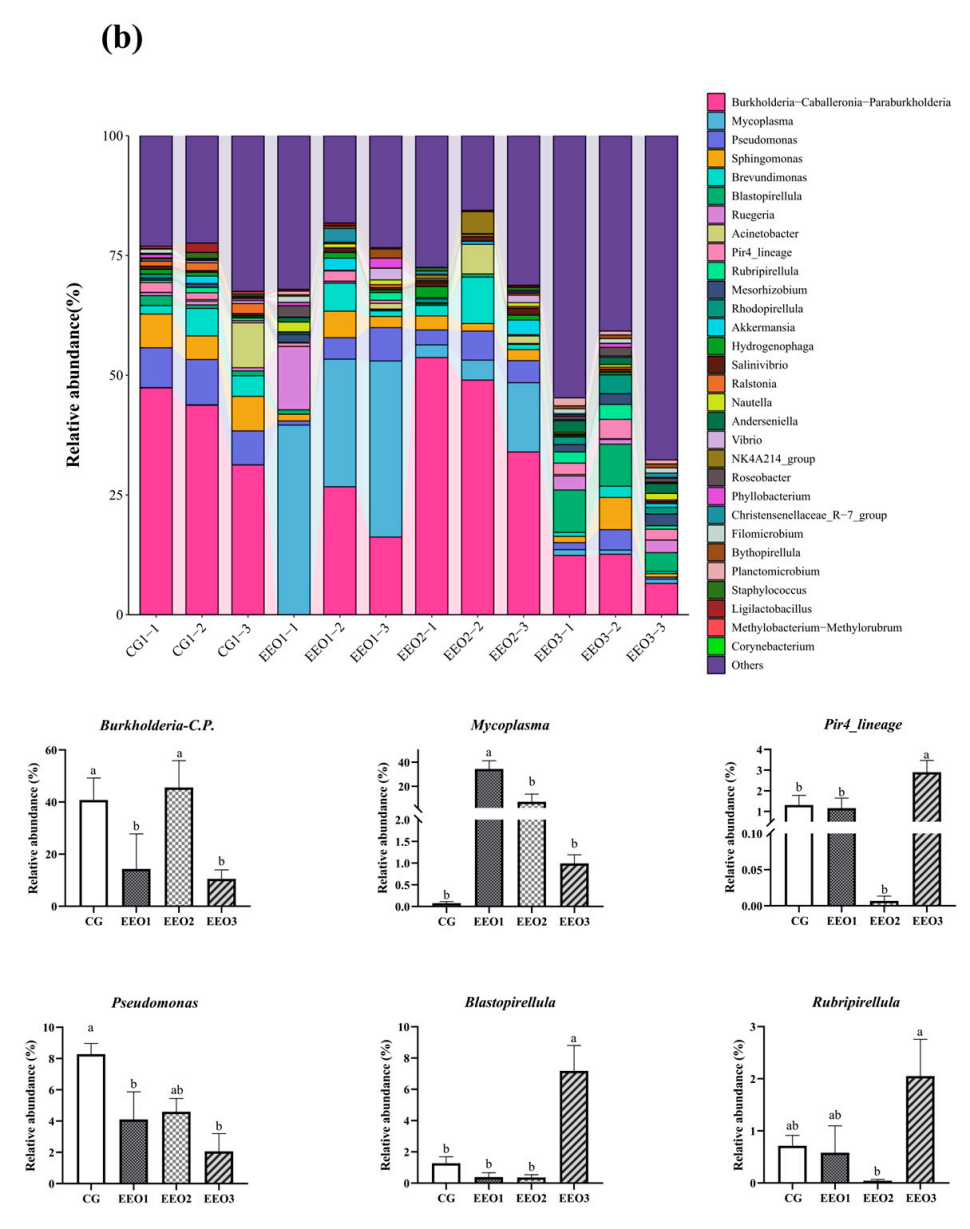
Intestinal microbial composition and relative abundance at various taxonomic levels: (**a**) phylum level (**b**) genus level. Each value represents mean ± SD (*n* = 3). Different letters above the bar represent significant differences among different groups (*p* < 0.05).

Figure 6 illustrates the proportion of microbiota across different taxonomic levels. At the phylum level, the top 10 phyla were counted and the proportion of *Proteobacteria*, *Firmicutes*, *Planctomycetota*, *Actinobacteria* and *Cyanobacteria* in these four groups accounted for 97.35%, 95.56%, 95.87% and 90.82%, respectively (Figure 6a). In addition, an analysis was conducted to examine the variations in abundance among the top ten phyla. In comparison to the CG group, there was a statistically significant reduction in the abundance of *Proteobacteria* within the EEO1 and EEO3 groups (*p* < 0.05). *Firmicutes* abundance in the EEO1 and EEO2 groups remarkably increased in comparison with the CG group (*p* < 0.05). The relative abundance of *Planctomycetota* and *Chloroflexi* in the EEO3 groups was also significantly higher than in the CG group (*p* < 0.05). At the genus level, the abundance of the top 30 genera and the differences among the top 10 phyla in abundance are presented in Figure 6b. The abundance of *Burkholderia-Caballeronia-Paraburkholderia* (*BCP*) and *Pseudomonas* significantly decreased in the EEO1 and EEO3 groups in comparison with the CG group (*p* < 0.05). The abundance of *Blastopirellula* and *Pire4_lineage* within the EEO3 group was obviously greater than that in the CG group (*p* < 0.05).

## 4. Discussion

Currently, the prevalence of microbial diseases acts as a major limiting factor for aquaculture. EOs have gained recognition as a potential dietary additive in the animal product field, particularly in aquatic animals because of their advantages in improving growth, antioxidative activity, immunity, tolerance to disease agents and gut microbiota [31,32,33,34].

In this study, in comparison with CG diets, EEO1 diets significantly promoted the WGR of *T. ovatus*. Similar research has found enhanced fish production when feeds incorporating various EOs are administered [35,36]. This parameter was significantly decreased in the EEO3 group, which suggested that an overly high concentration of EEO would inhibit growth. 1,8-cineole has a camphor-like and cooling herbal scent and is the main ingredient of eucalyptus EO [37], which may modify the flavor and reduce the palatability of dietary formulations when utilized in elevated concentrations. The promotion of growth in fish may be attributed to an enhanced ability to digest nutrients, and capacity for nutrient assimilation in fish depends on their intestinal morphology [38,39]. In the current study, significant increases were found in muscle layer thickness and the ratio of V/C, while obvious decreases in crypt depth were observed in the EEO2 group. An increase in intestinal muscle layer thickness can enhance the contractility of the intestine, making peristalsis smoother and helping to improve the intestinal capability to digest food and absorb nutrients [40]. Extended gut villi in fish are frequently associated with enhanced gastrointestinal health and increased nutrient assimilation efficiency, potentially resulting in a more robust digestive process [41]. The depth of the crypt reflects the ability of intestinal cells to differentiate villi and the V/C ratio has been recognized as a significant marker of digestive system health [42]. These findings suggested that administration of EEO could promote the growth of *T. ovatus* through improving their intestinal morphology.

Oxidative stress can arise from the excessive generation of reactive oxygen species (ROS), resulting in oxidative damage that negatively impacts fish well-being [43]. In fish, antioxidant substances (SOD, T-AOC and CAT) are commonly recognized as oxidative stress indicators, which play crucial roles in defense mechanisms against ROS [44,45]. MDA is a bioindicator reflecting lipid peroxidation, with a reduction in MDA concentrations associated with enhanced activity of antioxidative enzymes and/or an augmentation of non-enzymatic antioxidant defenses [46]. The present study showed that SOD and CAT activities obviously incremented and MDA level reduced in livers of fish fed the EEO1 diet. Similarly, dietary *Laurus nobilis* L. EO supplementation decreased oxidative stress in Nile tilapia [2]. EO derived from *Artemisia vulgaris* L. has the potential to mitigate intestinal disease in zebrafish by diminishing oxidative stress, inflammatory responses and tissue injury [45]. Antioxidant defenses are chiefly modulated via the nrf2/keap1 pathway [47]. The activation of nrf2 can upregulate antioxidant genes, allowing cells to produce more antioxidants to neutralize ROS, thereby reducing oxidative stress damage to cells [48]. Keap1, acting as a binding factor for nrf2, is responsible for recognizing and degrading nrf2 [49]. In our study, significantly upregulated expression of *nrf2*, *HO-1*, *GSH-Px*, *SOD*, *GR* and downregulated expression of *keap1* and *HSP70* were recorded in the EEO1 and EEO2 groups. GR, C4 and HO-1 are important immunological factors that play significant role in immune response through their antioxidant and anti-inflammatory properties [50,51,52]. These results imply that inclusion of a moderate EEO level can enhance antioxidative ability via the nrf2/keap1 pathway. The primary ingredient of eucalyptus EO is 1,8-cineole. Eucalyptus oil and 1,8-cineole have therapeutic uses in humans for upper respiratory tract infections, including anti-inflammatory, antibacterial, and biofilm reducing properties [53,54,55]. Previous studies have reported 1,8-cineole can confer protective benefits to fish against oxidative stress via enhancing antioxidative action [17,56], which was supported in our study. Interestingly, both antioxidant enzyme activities and gene expression levels showed abnormalities when fish were fed diets with EEO concentrations exceeding 5.0 mL/kg, indicating that an excessive EEO level may lead to an imbalance in the antioxidant system in fish, reducing their antioxidant capacity.

Intensive aquaculture practices frequently contribute to the incidence of diseases in fish populations. Streptococcosis is a term used to describe serious illnesses resulting from infections by Streptococcus species. This condition has been associated with an annual economic impact of approximately $150 million on the global aquaculture sector [57]. In this study, the bacterial load in five tissues of *T. ovatus* fed EEO2 diets exhibited obviously lower levels compared to those that were given the CG diets 24 h after challenge with *S. iniae B240703*, indicating that a moderate EEO level had great resistance against *Streptococcus iniae*. A decrease in disease prevalence would result in increased productivity and advantages for agriculture producers.

The homeostasis of intestinal microbes is linked to host health and is crucial for animal productivity, as it can help digest food, absorb nutrients, activate immune responses and resist disease [58]. In our study, the parameters of alpha diversity in the EEO3 group were found to be markedly greater than those in theother three groups, which suggested that EEO at high concentrations could alter the homeostasis of intestinal microbiota. Additionally, the beta diversity assessment revealed that juvenile *T. ovatus* among these groups have notably distinct communities, which may be attributed to the antibacterial characteristics of the EEO.

In this research, the predominant phyla identified were *Proteobacteria*, *Firmicutes*, *Actinobacteria* and *Cyanobacteria*, which aligns with our earlier investigations concerning the gut microorganisms of *T. ovatus* [3,59]. The proportion of *Proteobacteria* in the EEO-supplemented groups was less compared to that in the CG group. A significant abundance of *Proteobacteria* may play a role in disrupting the gut microbiota and eliciting esoenteritis [60]. *Firmicutes* are actively involved in the modulation of immune responses and the regulation of inflammatory symptoms via the synthesis of short-chain fatty acids [61]. The augmented proportion of *Firmicutes* in the EEO1 and EEO2 groups showed a moderate dietary EEO level was linked to positive outcomes in gut health. In addition, our study found that *Planctomycetota* within the EEO3 group exhibited a notably greater presence than that in other three groups. Previous reports found *Planctomycetota* to be a potential opportunistic pathogen existing in clinical specimens and hospital environments [62].

From the perspective of the genus level, the EEO1 group exhibited a reduced presence of *BCP* and *Pseudomonas*, alongside an increased proportion of *Mycoplasma*. Numerous species within the genera *BCP* and *Pseudomonas* have been established to cause disease in people and animals [63,64,65]. Previous investigation indicated that sialic acid lyase produced by *Mycoplasma* has the capacity to degrade sialic acids, thereby compromising the cell walls of pathogenic microbes. This mechanism serves to protect the host from infections [66]. These findings indicated that dietary EEO administration can maintain intestinal health by regulating the abundance of the dominant genus. Notably, the EEO3 group showed higher abundance of *Blastopirellula*, *Pir4_lineage* and *Rubripirellula* than the other three groups in this study. Previous studies found *Blastopirellula* was the predominant organism observed in infected seedlings of kelp [67,68]. The potential functions of *Pir4_lineage* and *Rubripirellula* had been not reported in fish. High concentrations of EEO altered the intestinal environment, which could lead to an increase in the abundance of these less common genera.

## 5. Conclusions

Our study showed that supplementing EEO improved the growth and intestinal morphology of *T. ovatus*. Inclusion of EEO enhanced antioxidative ability and immune function via the *nrf2*/*keap1* signaling pathway. Moreover, EEO promoted resistance against *Streptococcus iniae* and could help the aquaculture industry develop more sustainably. In addition, EEO has also been shown to optimize the composition of gut microbiota and regulate the diversity of genera present, thus maintaining the intestinal health of *T. ovatus*.

## Figures and Tables

**Table 1:** The compositions of the test diets.

Test Diets	Basic Diets ^1^ (g)	Eucalyptus Oil (mL)	Wine (mL)	Water (mL)
CG	100	0	3	3
EEO1	100	0.5	3	2.5
EEO2	100	1	3	2
EEO3	100	1.5	3	1.5

^1^ The basic diet was purchased from Guangdong Yuequn Marine Biotechnology Co., Ltd. The main nutritional levels of the dry matter are as follows: crude protein 40.0%, crude fat 6.0%, crude fiber 5.0%, crude ash 18.0%, lysine 2.1%, calcium 2.0%, total phosphorus 1.2%, moisture 10.0%.

**Table 2:** Targeted gene primer sequences used for qPCR analysis.

Genes		Sequence	Source
*β-Actin*	F	TGAACCCCAAAGCCAACAGG	[27]
	R	CCGCAGGACTCCATACCAAG	
*n* *rf2*	F	AGCTTGGCCTTCATCAAAT	[27]
	R	GAGTATGGCTGTCCTTCTTCA	
*k* *eap1*	F	CAGATAGACAGCGTGGTGAAGGC	[28]
	R	GACAGTGAGACAGGTTGAAGAACTCC	
*HO-1*	F	AGAAGATTCAGACAGCAGCAGAACAG	[28]
	R	TCATACAGCGAGCACAGGAGGAG	
*GSH-Px*	F	GCTGAGAGGCTGGTGCAAGTG	[28]
	R	TTCAAGCGTTACAGCAGGAGGTTC	
*HSP70*	F	TTGAGGAGGCTGCGCACAGCTTGTG	[29]
	R	ACGTCCAGCAGCAGCAGGTCCT	
*SOD*	F	CCTCATCCCCCTGCTTGGTA	[28]
	R	CCAGGGAGGGATGAGAGGTG	
*C4*	F	TGGAGAAAAAGTTAAAGGGGC	[29]
	R	CAGGAAGGAAGTATGAGCGAGT	
*GR*	F	GTGTGTGTGGGCAAGGAGGA	[29]
	R	AGATGAGGTGGGGTGAATGG	

*nrf2*: nuclear factor erythroid 2-related factor 2, *keap1*: kelch-like ECH-associated protein 1, *HO-1*: heme oxygenase 1, *GSH-Px*: glutathione peroxidase, *HSP70*: heat shock protein, *SOD*: superoxide dismutase, *C4*: Complement 4, *GR*: glutathione reductase.

**Table 3:** Effects of dietary EO levels on intestinal alpha diversity in *T. ovatus*.

Parameters	CG (0)	EEO1 (0.5%)	EEO2 (1.0%)	EEO3 (1.5%)
Observed_species	272.67 ± 12.22 ^b^	186.67 ± 42.22 ^b^	196.00 ± 63.22 ^b^	428.00 ± 76.62 ^a^
Shannon	4.81 ± 0.31 ^b^	4.10 ± 0.38 ^b^	4.12 ± 0.63 ^b^	6.84 ± 0.29 ^a^
Simpson	0.86 ± 0.04 ^b^	0.86 ± 0.02 ^b^	0.82 ± 0.06 ^b^	0.98 ± 0.00 ^a^
Chao1	276.18 ± 12.93 ^b^	190.77 ± 41.14 ^b^	197.34 ± 64.06 ^b^	431.08 ± 74.22 ^a^
Goods_coverage	1.00 ± 0.00	1.00 ± 0.00	1.00 ± 0.00	1.00 ± 0.00
Pielou_e	0.59 ± 0.03 ^b^	0.54 ± 0.04 ^b^	0.55 ± 0.08 ^b^	0.79 ± 0.02 ^a^
Ace	276.74 ± 11.75 ^b^	191.42 ± 40.68 ^b^	198.50 ± 64.61 ^b^	432.42 ± 74.92 ^a^

Values in each row with different superscript letters are significantly different (*p* < 0.05).

## Data Availability

The original contributions presented in this study are included in the article/Supplementary Material. Further inquiries can be directed to the corresponding authors.

## Supplementary Materials

The following supporting information can be downloaded at: https://www.mdpi.com/article/10.3390/microorganisms13030537/s1, Table S1. Targeted gene primer sequences used for qPCR analysis.
