# Supplementary material for: Effects of Eucalyptus Essential Oil on Growth, Immunological Indicators, Disease Resistance, Intestinal Morphology and Gut Microbiota in Trachinotus ovatus
Source: Microorganisms. 2025 Feb 27;13(3):537. doi: 10.3390/microorganisms13030537 (PMC11944555; doi:10.3390/microorganisms13030537)
Supplement: Supplementary file 1 [file microorganisms-13-00537-s001.zip › microorganisms-3453575-supplementary.pdf]

SUPPLEMENTARY MATERIALS

Methods of qPCR

RT-qPCR was performed in a reaction volume of 12.5 μL containing 6.25 μL SYBR® Premix Ex Taq™ II (2 ×) (TaKaRa), 1 μL cDNA template, 0.5 μL each forward and reverse primers and 4.25 μL DEPC-treated water. The thermal profile for qPCR was 95°C for 30 s, followed by 40 cycles of 95°C for 10 s, and then 60°C for 30 s. The assay of each mixed sample was repeated three times. Gene expression levels were quantified relative to the expression of β-actin according to the $2^{-\Delta\Delta Ct}$ method.

Table S1. Targeted gene primer sequences used for qPCR analysis

Genes		Sequence
gyrB	F	GAAGATGATTCCATTACCGTTG
	R	CTAAATCTTCTCCTACCACACC
